# Supplementary material for: Neurodevelopmental Changes in the Guinea Pig Brain Caused by Time-Limited Complete Vitamin C Deprivation
Source: Nutrients. 2025 Nov 6;17(21):3484. doi: 10.3390/nu17213484 (PMC12611027; doi:10.3390/nu17213484)
Supplement: Supplementary file 1 [file nutrients-17-03484-s001.zip › nutrients-3931783-supplementary.pdf]

Table S1. Distribution of fetuses according to the analysis

	Control group										E1 Vitamin C-deprived group										E2 Vitamin C-deprived group									
**Dam number**	**1**	**2**	**3**	**4**	**5**	**6**	**7**	**8**	**9**	**10**	**1**	**2**	**3**	**4**	**5**	**6**	**7**	**8**	**9**	**10**	**1**	**2**	**3**	**4**	**5**	**6**	**7**	**8**	**9**	**10**
**Number of fetuses**	3	4	3	3	3	3	4	3	3	3	3	3	4	3	4	4	3	3	3	3	3	3	3	3	4	3	3	3	3	3
**Biochemical analysis**	1	1	1	1	1	1	-	-	-	-	1	1	1	1	1	1	-	-	-	-	1	1	1	1	1	1	-	-	-	-
**Molecular analysis**	1	1	1	1	1	1	-	-	-	-	1	1	1	1	1	1	-	-	-	-	1	1	1	1	1	1	-	-	-	-
**Histology analysis**	1	2	1	1	1	1	4	3	3	3	1	2	1	1	2	2	1	3	1	1	1	1	1	1	2	1	3	3	3	3
